# A Case of Fulminant Myocarditis With Preceding Repeated Episodes of Congestive Heart Failure

**DOI:** 10.4021/cr261w

**Published:** 2013-05-09

**Authors:** Yuko Tada, Kenta Uto, Hiroshi Wada, Ken-ichi Sakakura, Jun-ichi Suzuki, Toshio Nishikawa, Junya Ako, Shin-ichi Momomura

**Affiliations:** a Division of Cardiovascular Medicine, The University of Tokyo, Japan; b Department of Pathology, Tokyo Women’s Medical University, Japan; c Department of Cardiovascular Medicine, Saitama Medical Center, Jichi-Medical University, Japan; d Department of Advanced Clinical Science and Therapeutics, University of Tokyo, Japan; e Department of Surgical Pathology, Tokyo Women’s Medical University, Japan

**Keywords:** Myocarditis, Heart failure, Episode

## Abstract

We report a rare case of fulminant myocarditis that was considered to have smoldered for a few months before it finally exteriorized. An 80-year-old man had had two episodes of mild congestive heart failure with preserved ejection function (HFPEF) within 3 months before he was finally admitted for the treatment of rapidly progressive heart failure. Cardiac function deteriorated remarkably on the final admission. Extracorporeal cardiopulmonary support was used because of pump failure and conduction disability, however, the patient died on the 16th day. Endomyocardial biopsy revealed numerous inflammatory infiltrates in myocardium compatible with fulminant myocarditis. However, advanced fibrosis and increased number of B lymphocytes and plasma cells found in the present case were not typical for fulminant myocarditis. Considering several distinctive findings in clinical and laboratory findings together, two preceding HFPEF episodes were highly likely to be associated with myocarditis.

## Introduction

Myocarditis, while its pathogenesis has not yet been fully understood, develops in various ways, including acute, fulminant and chronic myocarditis and is sometimes diagnosed incidentally as the pathogenesis of dilated cardiomyopathy or arrhythmia [1]. In fact, particularly active types of myocarditis which progress over months or years and lead to death have been also reported [2-4]. However, even in those active types of myocarditis, myocarditis which finally develops fulminant myocarditis has never been reported.

We report a case of fulminant myocarditis that seemed to smolder for a few months. He had been on treatment for heart failure with preserved ejection fraction (HEPEF) that repeated twice during 3 months when he was finally diagnosed with fulminant myocarditis. It seemed highly likely that the preceding HFPEFs were caused in the course of myocarditis, reviewing several distinctive factors in clinical, laboratory and histological findings.

## Case Report

An 80 year-old male was admitted for the treatment of worsening dyspnea and chest pain. He had had two episodes of HFPEF within 3 months prior to this admission. He first noticed mild chest discomfort at rest in December 2009. Troponin T test was positive. Electrocardiography showed frequent premature ventricular contractions (PVCs). Global left ventricular (LV) function was preserved on echocardiography except for slight hypokinesis in basal posterior wall with an ejection fraction of 54%. Pseudonormalized LV inflow wave pattern (E/A ratio 1.6 and deceleration time 290 ms) were compatible with impaired diastolic function. Though coronary angiography (CAG) revealed total occluded lesion in the proximal portion of right coronary artery, there was a good collateral flow from left anterior descending artery. Mild congestive heart failure (CHF) was diagnosed with elevated plasma brain natriuretic peptide (BNP) level (991 pg/mL). He was discharged with blocker. One month later, he was admitted due to recurrence of CHF with elevated plasma BNP level of 1,319 pg/mL. Creatinine kinase (CK) was not elevated. Findings of electrocardiography and cardiac function evaluated by echocardiography were similar to the previous ones. At that time, diuretics were added.

**Figure 1:**
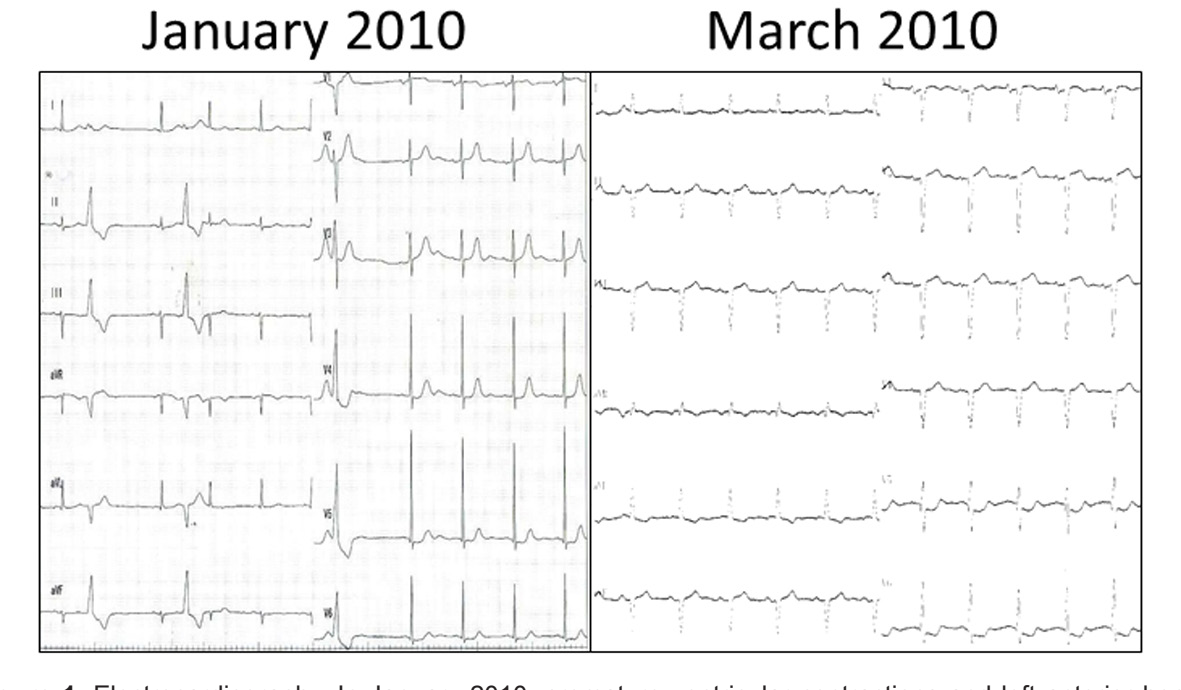
Electrocardiography. In January 2010, premature ventricular contractions and left anterior hemiblock were seen (Left). On admission in March 2010, conduction disturbance was suggested from wide QRS and Q wave in multiple leads appeared (Right).

Finally, in the middle of February 2010, he was admitted for the third time due to relapsing heart failure. He complained of irritable chest pain aggravated by breathing or changing positions and had low grade fever lasting for a few days. Electrocardiographic changes, such as wide QRS and abnormal Q waves in inferior and prechordal leads appeared (Fig. 1). Echocardiography also showed remarkable changes; cardiac function was impaired with 40% of LV ejection fraction. Laboratory findings showed elevated BNP level of 2,419 pg/mL, CK of 282 IU/L and CRP of 7.82 mg/dL. Acute coronary syndrome was considered less likely, since urgent CAG showed no new lesions of obstruction or stenosis and thallium scintigraphy revealed no perfusion defects at rest. This time, endomyocardial biopsy from right ventricular septum was performed. Percutaneous cardio-pulmonary support device (PCPS), intra artery balloon pumping (IABP) and transvenous pacing were inserted on the 4th hospital day because of advanced atrioventricular block (AVB) and uncontrollable ventricular tachycardia (VT). After PCPS and IABP were weaned off, he died of sudden cardiac arrest on the 16th hospital day. Autopsy was not performed.

**Figure 2:**
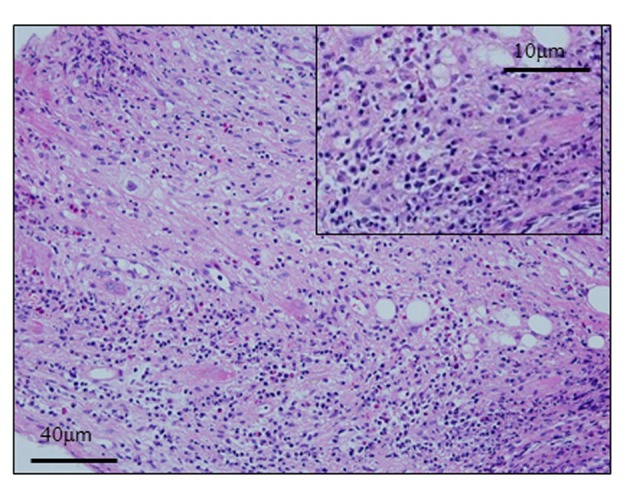
Hematoxylin- Eosin staining. Endomyocardial biopsy was performed from right ventricular septum on the 4th hospital day. The biopsied specimen was formalin fixed and paraffin embedded, (square: high power field).

**Figure 3:**
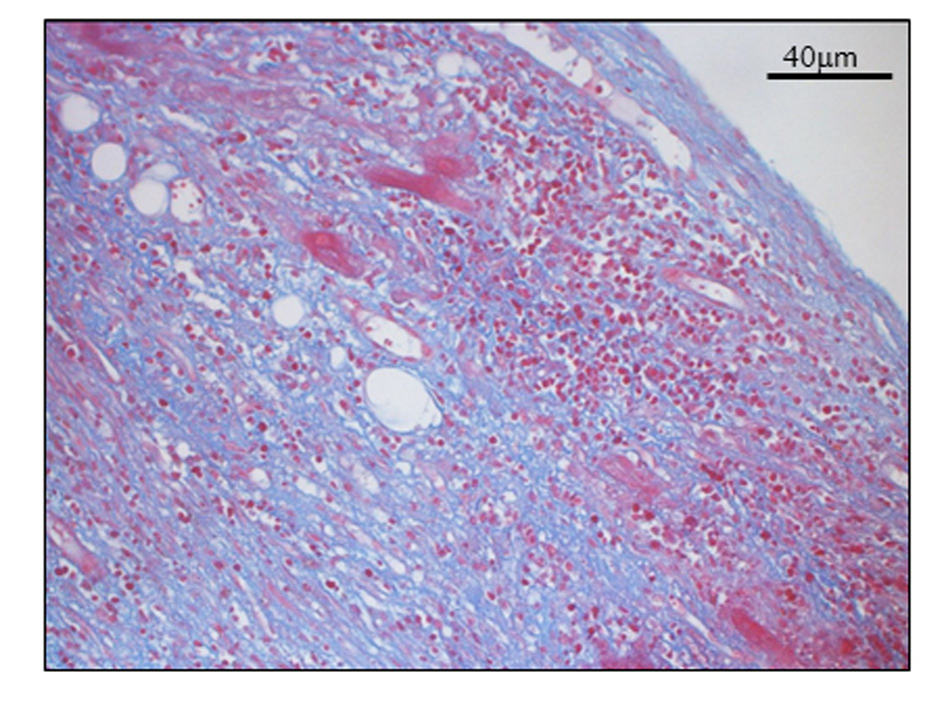
Azan staining. Azan staining showed extensive fibrosis.

**Figure 4:**
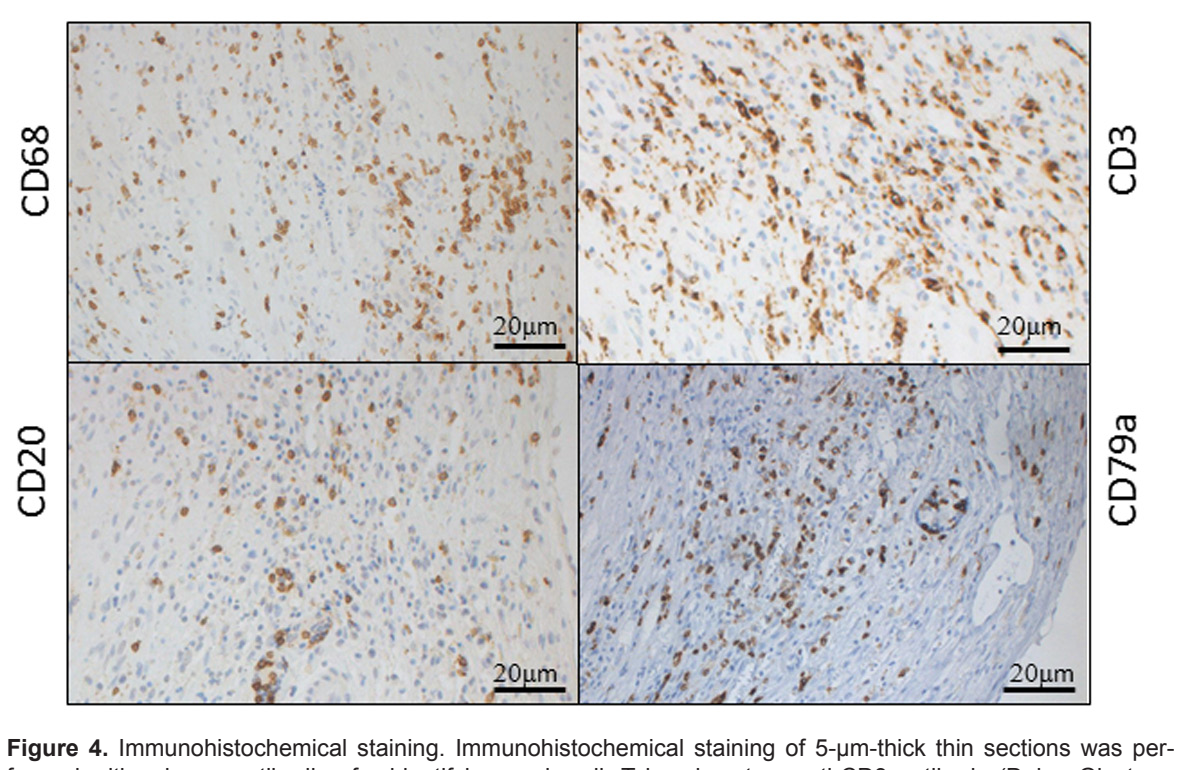
Immunohistochemical staining. Immunohistochemical staining of 5-µm-thick thin sections was performed with primary antibodies for identifying each cell; T lymphocytes: anti-CD3 antibody (Dako, Glostrup, Denmark), B lymphocytes and Plasma cells: anti-CD20 and anti-CD79a antibodies (Dako), monocytes and macrophages: anti-CD68 (Dako). Incubation with an avidin-biotin-blocking system and secondary antibody and peroxidase-labeled avidin-biotin complex system (Dako) was performed then. Localization of the primary antibody was visualized with 3, 3’ diaminobenzidine (DAB), followed by counterstaining with hematoxylin. Upper left: CD68, lower left: CD20, lower right: CD79a, upper right: CD3

Histological findings of Hematoxylin-Eosin staining and azan staining are shown in Figure 2 and 3. Myocardium was destroyed vastly. Fibrosis was diffuse and severe. Inflammatory cells mainly composed of lymphocytes, plasma cells and macrophages, infiltrated extensively in interstitium. They were identified with immunohistochemical staining. Macrophages identified as CD68 positive cells were most frequently found. Among lymphocytes, B lymphocytes or plasma cells represented by CD20 or CD79a positive cells were frequently found, while CD 3 positive T lymphocytes were relatively fewer (Fig. 4). Based on acute hemodynamic collapse and histological findings, he was diagnosed with fulminant myocarditis. Advanced fibrosis and B-cell dominant population of lymphocytes were not typical for fulminant myocarditis but rather suggestive of myocarditis persisting for a longer period of time.

## Discussion

We experienced a case of fulminant myocarditis that seemed to have been smoldering for 3 months with repeated history of heart failure with preserved ejection fraction (HFPEF). Myocarditis which evokes recurrent HFPEF and finally causes fulminant myocarditis has not been reported.

Although we could not confirm diagnosis of myocarditis without endomyocardial biopsy in the preceding episodes of HFPEF, it seemed most likely that the whole events were the course of myocarditis. First, it may be reasonable to think that recurrent episodes of heart failures in relatively short periods of 3 months were related to the final episode of fulminant myocarditis. Second, HFPEF of the present case had some atypical characteristics. He did not have common features of HFPEF, such as hypertension or left ventricular hypertrophy. Though he had a coronary artery disease, it was a chronic lesion, supported by well-advanced collateral arteries and seemed unlikely as the cause of treatment-resistant heart failure. It would be uncommon to show rather high plasma BNP and positive troponin T test if this patient was simply a case of HFPEF [5]. Finally, histological findings showed some atypical features of acute phase of fulminant myocarditis that have been reported, as described below.

Preceding episodes of heart failure are atypical for fulminant myocarditis. Fulminant myocarditis is defined as lethal acute myocarditis entailing severe heart failure and collapsing of hemodynamics, first described by Fiedler in 1899 [6, 7]. Pathological findings are characterized by the active inflammatory infiltrate and multiple foci of myocyte necrosis. Histological classification, including lymphocytic myocarditis, giant cell myocarditis and eosinophilic myocarditis is already established. In general, fulminant myocarditis is distinguished from acute myocarditis or chronic myocarditis clinically: fulminant myocarditis is indicated by distinct onset with prodromal symptoms of viral infection, acute and severe LV dysfunction, almost complete recover only with supportive therapy and so on [2, 3]. In contrast, acute myocarditis and chronic myocarditis have indistinctive onset and rather poor prognosis [2, 3]. Fulminant myocarditis which smoldered as the present case has never been reported.

Characteristics of lymphocytes found in endomyocardial biopsy in the present case were considered atypical for acute myocarditis or acute phase of fulminant myocarditis. B lymphocytes or plasma cells were more frequently found than T lymphocytes in the present case, although T lymphocytes and few B lymphocytes were generally found in the very acute phase of myocarditis. Because we performed endomyocardial biopsy shortly after the last recurrence of heart failure, it was suggested that myocarditis started in rather earlier time point. In fact, according to the past reports of some human cases and animal models of myocarditis, T lymphocytes infiltrate mainly in fulminant myocarditis or acute myocarditis, followed by infiltration of B lymphocytes in chronic phase [8, 9]. This should reflect the fact that the shift of inflammation from acute into chronic state often parallels the shift of immunological balance from Th1 (or cellular mediated immunity) into Th2 (or humoral mediated immunity). However, diagnostic usefulness of these findings has not been established, since the pathogenesis of myocarditis should be complex depending on the association between immunological background of hosts and pathogens, as elicited in models of some mouse strains [10, 11].

The present case seems to have several similarities with fatal and progressive type of chronic myocarditis described by Fenoglio et al as ‘rapidly-progressive’ myocarditis in 1983 [4]. He divided myocarditis into three types; acute myocarditis, ‘rapidly-progressive’ myocarditis and chronic myocarditis based on histological and clinical findings. Clinical findings of rapidly-progressive myocarditis were characterized by alternating episodes of intractable heart failure and remission. Cardiac arrhythmias could often be a major problem. The typical histological findings are numerous foci of cell damage and extensive fibrosis. Lieberman et al. also described the similar type of myocarditis as chronic active myocarditis [3]. Both of them reported this type of myocarditis finally led to severe LV dysfunction and fatal outcome within several months or years. Myocarditis of the present case might possibly fall in the category of ‘rapidly-progressive’ myocarditis, judging from several similarities in clinical and histological findings.

In summary, we described a rare case of fulminant myocarditis which repeated HFPEF before it finally exteriorized. The present case is important in suggesting that fulminant myocarditis could smolder and entail preceding episodes of heart failure. Histological findings including immunohistochemical findings should be particularly important in diagnosing myocarditis, especially in complex cases.
